# Geographic Disparities in Mortality Risk Within a Racially Diverse Sample of U.S. Veterans with Traumatic Brain Injury

**DOI:** 10.1089/heq.2018.0047

**Published:** 2018-10-25

**Authors:** Clara E. Dismuke-Greer, Mulugeta Gebregziabher, Tiarney Ritchwood, Mary Jo Pugh, Rebekah J. Walker, Uche S. Uchendu, Leonard E. Egede

**Affiliations:** ^1^ Health Equity and Rural Outreach Innovation Center, Ralph H. Johnson Veterans Affairs Medical Center, Charleston, South Carolina.; ^2^ Division of Internal Medicine, Department of Medicine, Medical University of South Carolina, Charleston, South Carolina.; ^3^ Department of Public Health Sciences, Medical University of South Carolina, Charleston, South Carolina.; ^4^ Department of Internal Medicine, University of Utah School of Medicine, Salt Lake City, Utah.; ^5^ IDEAS Center of Innovation, VA Salt Lake City Health Care System, Salt Lake City, Utah.; ^6^ Division of General Internal Medicine, Department of Medicine, Medical College of Wisconsin, Milwaukee, Wisconsin.; ^7^ Center for Advancing Population Science (CAPS), Medical College of Wisconsin, Milwaukee, Wisconsin.; ^8^ Chief Officer for Health Equity, US Department of Veterans Affairs, Washington, DC.; ^9^ Principal, Health Management Associates, Washington, DC.

**Keywords:** traumatic brain injury, veteran, mortality, geographic, disparities, racial/ethnic

## Abstract

**Purpose:** Traumatic brain injury (TBI) is a signature injury among the U.S. veterans. Hispanic U.S. veterans diagnosed with TBI have been found to have higher risk-adjusted mortality. This study examined the adjusted association of geographic location with all-cause mortality in 114,593 veterans diagnosed with TBI between January 1, 2000 and December 31, 2010, and followed through December 31, 2014.

**Methods:** National Veterans Health Administration (VHA) databases containing administrative data including *International Classification of Diseases, 9th Revision* (*ICD-9*) codes, sociodemographic characteristics, and survival were linked. TBI was identified based on *ICD-9* codes. Cox proportional hazards regression methods were used to examine the association of time from first TBI *ICD-9* code to death with geographic location, after adjustment for TBI severity, race/ethnicity, other sociodemographic characteristics, military factors, and Elixhauser comorbidities.

**Results:** Relative to urban mainland veterans with a median survival of 76.4 months, veterans living in the U.S. territories had a median survival of 69.1 months, whereas rural mainland veterans had a median survival of 77.1 months, and highly rural mainland veterans had a mean survival of 77.6 months. The final model adjusted for race/ethnicity, TBI severity, sociodemographic, military, and comorbidity covariates showed that residing in the U.S. territories was associated with a higher risk of death (hazard ratios=1.24; 95% confidence interval 1.15–1.34) relative to residing on the U.S. mainland. The race/ethnicity disparity previously found for the U.S. veterans diagnosed with TBI seems to be accounted for by living in the U.S. territories.

**Conclusion:** The study shows that among veterans with TBI, mortality rates were higher in those who reside in the U.S. territories, even after adjustment. Previous documented higher mortality among Hispanic veterans seems to be explained by residing in the U.S. territories. The VA has a mission of ensuring equitable treatment of all veterans, and should investigate targeted policies and interventions to improve the survival of the U.S. territory veterans diagnosed with TBI.

## Introduction

Traumatic brain injury (TBI) is a mild, moderate, or severe disruption in the normal functioning of the brain caused by a penetrating head injury or bump, blow, or jolt to the head. TBI is diagnosed in >1.7 million people in the United States each year.^1,2^ It is among the leading causes of death and disability in the United States, accounting for 30% of all injury-related deaths.^3^ There has been an increasing amount of research on TBI focused on military personnel, as >360,000 U.S. veterans across all branches of the military have been diagnosed with TBI since the year 2000.^4^ The U.S. veterans are 1.5 times more likely than their civilian counterparts to die owing to complications from TBI, leading to life expectancies that are reduced by 4 years.^5^ In addition to higher mortality rates for more severe TBI, a large proportion of military personnel suffer from mild TBI.^6^ TBI has been particularly devastating for veterans deployed to Iraq and Afghanistan as part of Operation Iraqi Freedom (OIF) and Operation Enduring Freedom (OEF), with nearly 20% experiencing a mild TBI.^7^ Although symptoms of mild TBI typically resolve within 3 months, many veterans experience long-term complications and disability, in addition to comorbid mental health challenges, including post-traumatic stress disorder.^8^ Additional concerns include higher health care costs associated with caring for individuals suffering from TBI.^9^ For example, Veterans with TBI have health care costs that are four times higher than their peers without TBI.^10^ Given the burden of TBI among veterans, more efforts are needed to identify individual and environmental characteristics that potentially contribute to elevated mortality rates.

One patient characteristic often associated with TBI-related mortality among veterans is race/ethnicity. Previous research using national Veterans Administration (VA) data showed Hispanic ethnicity was associated with higher mortality risk among veterans clinically diagnosed with TBI.^10,11^ In addition, although Hispanic veterans are more likely than their non-Hispanic White peers to have greater TBI severity, they are less likely to utilize TBI services, including specialty clinics, neurology, and rehabilitation services in the VA health care system.^10,12^ Ethnic and racial disparities in health care are particularly concerning in the VA health care system, which aims to be an equal access network of government-administered, comprehensive health care facilities that serves >9 million veterans, with ∼22% being individuals from racial or ethnic minority backgrounds.^13^

Although there are a number of potential reasons for racial and ethnic disparities in TBI-related mortality among veterans, including age, injury severity, health literacy, and practice variations (i.e., when patients with similar diagnoses and medical histories receive unequal levels of care depending on when, where, and by whom they are treated), emerging evidence suggests that geographic location may significantly impact health outcomes.^13^ The research on this association, however, is sparse. One study using data from MarketScan—a database containing individual-level information on clinic usage, health care costs, from employers, health plans, and government and public organizations—found TBI mortality was higher in the Midwest and Southeastern regions of the United States.^15^ The states with the highest mortality rates were Mississippi, North Carolina, Kansas, and Idaho. The states with the lowest mortality rates were Alabama, Arkansas, Connecticut, and Oregon. States in the Western region of the United States had the lowest rates of TBI mortality (e.g., California and Washington). Previous research on other chronic conditions has also found geographic variations in health management and outcomes. Specifically, patients in rural areas have been found to have worse outcomes in cardiovascular disease,^16,17^ chronic obstructive pulmonary disease,^18^ and chronic lower leg wound care.^19^ The results of another study suggested that elder veterans residing in rural areas were less likely than their urban peers to receive quality health care as measured by the quality of medication prescribing.^20^ However, it is unknown whether health outcomes, specifically TBI-related mortality, differ by region or between veterans living in the U.S. mainland and U.S. territories.

Taken together, the results of previous research highlight the importance of race and ethnicity in TBI-related mortality among veterans; however, it is unclear whether racial and ethnic disparities are influenced by geographic location. As veterans eligible for benefits do not pay premiums, the VA health care system provides a unique opportunity to study the determinants of health care disparities within this population without being confounded by financial barriers that determine whether an individual can afford medical treatment, a serious limitation of the private insurance sector. Therefore, the purpose of this study was to examine the longitudinal associations among race and ethnicity, geographic location, and all-cause mortality in veterans with TBI.

## Methods

### Procedures

We identified 114,593 veterans with clinically diagnosed TBI (diagnosed at any time) based upon *International Classification of Diseases, Ninth Revision* (*ICD-9*) codes TBI, postconcussive syndrome, and TBI-related late effects mandated by the VA that were seen at VA medical centers and community-based outpatient clinics between January 1, 2000 and December 31, 2010 and followed through December 31, 2014.^21^ Next, we linked multiple patient and administrative files from VA Informatics and Computing Infrastructure (VINCI) databases—the Veterans Health Administration (VHA) Decision Support System, and Patient Treatment and Vital Status files—using scrambled social security number to create a national cohort of veterans with TBI. The VHA Decision Support System is a national automated management information system that uses commercial software to integrate data from clinical and financial systems for inpatient and outpatient veteran care. All study procedures followed a protocol approved by the local Institutional Review Board and VA Research and Development Committee.

### Measurements

#### Clinically diagnosed TBI

Veterans with clinician-confirmed diagnoses of TBI were included in this study. Veterans with clinically diagnosed TBI were identified based on having an *ICD-9* code for TBI in an inpatient hospitalization or outpatient encounter of 310.2, 850.xx, 851.xx, 852.xx, 853.xx, 854.xx, 800.xx, 801.xx, 803.xx, 804.xx, 950.1x, 907.0, 959.01, and V15.xx per Military Health System and The Defense Health Agency 2016 guidelines.^21^ TBI severity was also classified per the Military Health System and the Defense Health Agency (2016) guidelines as mild, moderate, severe, penetrating, or unknown based on *ICD-9* codes and the guidelines.^21^

#### Sociodemographics

We collected sociodemographic and clinical variables from the VINCI patient treatment and vital status files. Date of death was obtained from the vital status file between January 1, 2000 and December 31, 2014. Time to death was defined as the number of months from time of entry into the study based on first identified TBI *ICD-9* code, after January 1, 2000 until time of death or end of study censoring (December 31, 2014). Race/ethnicity was based upon self-report and classified as non-Hispanic White, non-Hispanic Black, Hispanic, and Other race. Age was categorized into four groups (18–34, 35–50, 51–61, 62 years and older). Gender was classified as a binary indicator with female=1. OEF or OIF service was classified as a binary indicator with history of service=1. Service in combat was classified as a binary indicator with any reported combat service in OEF or OIF conflicts=1. Service connected disability was classified as a binary indicator with any reported service connected disability=1. Marital status was classified as a binary indicator of married=1. Veteran location was based on zip-code for all but veterans residing in the U.S. territories. The U.S. Department of Veterans Affairs Office of Rural Health defines rurality based on the U.S. Department of Agriculture (USDA), and U.S. Department of Health and Human Services (HHS) developed Rural-Urban Commuting Areas (RUCA) system.^22^ The RUCA system accounts for population density and the socioeconomic proximity of a community to larger urban centers.^22^ RUCA is also based on citizen counts by the U.S. Census Bureau.^22^ According to RUCA, an urban area consists of census tracts with at least 30% of the population residing in an urbanized area.^22^ A highly rural area is a sparsely populated area with <10% of the working population commuting to any community larger than an urbanized cluster, generally a town whose population does not exceed 2500 people.^22^ A rural area is a land area that does not fall into the category of urban or highly rural area.^22^

Veterans residing in the U.S. territories without a zip-code were classified based on the VA medical center or community-based outpatient clinic where they received care. The U.S. Territories included Puerto Rico, U.S. Virgin Islands, Guam, America Samoa, and Mariana Islands.

#### Comorbid conditions

We classified the following 31 Elixhauser comorbidities as binary indicators based on *ICD* codes identified by definitions of Quan et al.^23^: congestive heart failure, cardiac arrhythmias, valvular disease, pulmonary circulation disorders, peripheral vascular disorders, uncomplicated hypertension, complicated hypertension, paralysis, other neurological disorders, chronic pulmonary disease, uncomplicated diabetes, complicated diabetes, hypothyroidism, renal failure, liver disease, peptic ulcer disease excluding bleeding, AIDS/HIV, lymphoma, metastatic cancer, solid tumor without metastasis, rheumatoid arthritis/collagen vascular diseases, coagulopathy, obesity, weight loss, fluid and electrolyte disorders, blood loss anemia, deficiency anemia, alcohol abuse, drug abuse, psychoses, and depression.

### Analysis

We conducted a series of preliminary analyses. First, we examined the unadjusted associations between survival in months and veteran geographic location of residence using the Kruskal–Wallis equality-of-populations test. Next, we examined the unadjusted association between mortality and a binary indicator of death using chi-square test. We then examined the unadjusted associations between veteran geographic location, race/ethnicity, other sociodemographic characteristics, military variables, and TBI severity, using chi-square test.

We then used Cox proportional hazards regression methods to examine the association of time to death with geographic location after checking for the proportional hazards assumption through covariate-by-log(time) interaction. For all veterans diagnosed with TBI, we created four models to provide hazard ratios (HRs) for mortality risk and their corresponding 95% confidence intervals (CIs). Given the previous evidence of the association of Hispanic ethnicity with mortality among veterans diagnosed with TBI, we adjusted the first model for race/ethnicity only, the second model for location only, the third model for race/ethnicity and location, and the fourth model for all sociodemographic characteristics, military factors, TBI severity, and binary indicators of Elixhauser comorbidities. We also fitted a model with the binary indicators of comorbidities replaced with count of comorbidities. We used the Kaplan–Meier method to plot the survival function. We assessed the fit of the model using residual analysis and all analyses were conducted using STATA version 14.0 in VINCI.

## Results

Table 1 gives the frequency of geographic location. Most (68.5%) of the veterans diagnosed with TBI resided in mainland urban areas, with 27.8% in mainland rural areas. More veterans (2.5%) resided in the U.S. territories than in mainland highly rural areas (1.2%). Table 1 also gives median survival in months, death rates, and categorical indicators of sociodemographic, military, TBI severity, and median number of comorbidities by geographic location. Relative to urban mainland veterans diagnosed with TBI with a median survival of 76.4 months, veterans living in the U.S. territories had a median survival of 69.1 months, whereas mainland rural veterans had a median survival of 77.1 months, and highly rural mainland veterans had a median survival of 77.6 months (*p*=0.0001). Relative to 20.9% mortality for urban mainland veterans diagnosed with TBI, 36.7% of the U.S. territory veterans died during the study period, whereas 20.5% of rural mainland and 19.6% of highly rural mainland veterans died. Race/ethnicity, TBI severity, age, gender, OEF/OIF status, combat status, service connected disability status, marital status, and median number of Elixhauser comorbidities showed significant differences by veteran location. Veterans who resided in the U.S. territories had the highest percentage of Hispanic race/ethnicity (64.6%), moderate TBI (72.1%), and median number of Elixhauser (6.0) comorbidities.

**Table 1.** **Sample Characteristics by Geography: Geographic Disparities in the U.S. Veteran Traumatic Brain Injury Mortality, January 1, 2000 to December 31, 2014**

	Mainland urban, *N*=78,556 (68.5%)	Mainland rural, *N*=31,860 (27.8%)	Mainland highly rural, *N*=1306 (1.2%)	U.S. territories, *N*=2872 (2.5%)
Survival in months (median)	76.4	77.1	77.6	69.1
Died^a^, %	20.9	20.5	19.6	36.7
Race/ethnicity^a^, %				
Non-Hispanic White	73.6	89.4	90.5	25.4
Non-Hispanic Black	16.8	5.5	2.2	3.0
Hispanic	6.1	2.2	1.8	64.6
Other race	3.5	2.9	5.5	6.9
TBI severity^a^, %				
Mild TBI	24.8	26.0	24.7	22.1
Moderate TBI	58.9	56.2	55.2	72.1
Severe TBI	5.0	6.0	5.6	3.0
Penetrating TBI	0.9	0.8	0.8	1.0
Unknown TBI severity	10.4	11.0	13.6	1.7
Age categories^a^				
Age 18–34, %	30.2	28.1	23.8	13.4
Age 35–50, %	24.2	23.3	19.9	17.3
Age 51–61, %	22.3	23.5	26.7	16.8
Age 62 and older, %	23.2	25.1	29.6	52.4
Female^a^, %	7.1	5.5	4.7	4.0
OEF/OIF veteran^a^, %	30.5	31.2	26.4	18.5
Combat veteran^a^, %	26.3	27.2	23.9	18.0
Any service connected disability^a^, %	43.4	45.2	44.6	41.0
Married^a^, %	40.6	51.2	53.5	56.6
Median number of Elixhauser comorbidities^a^	4.0	4.0	4.0	6.0

The sample size was 114,593 veterans with an *ICD-9* code for TBI served by the Veterans Health Administration between January 1, 2000 and December 31, 2010 and followed until death or December 31, 2014. U.S. Territories include Puerto Rico, U.S. Virgin Islands, Guam, America Samoa, and Mariana Islands.

^a^ *p*<0.05.

*ICD-9, International Classification of Diseases, 9th Revision*; OEF, Operation Enduring Freedom; OIF, Operation Iraqi Freedom; TBI, traumatic brain injury.

**FIG. 1.**
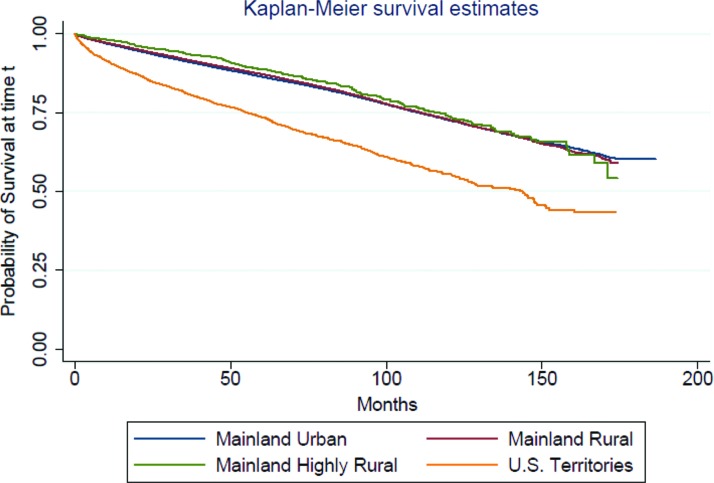
Kaplan–Meier survival curve for time to death by veteran geographic location.

We created four Cox proportional hazard regression models to examine the effect of specific sets of covariates on the association between risk of mortality and location of residence among veterans clinically diagnosed with TBI. Table 2 provides the HRs and 95% CIs for the sequentially built models. The first model, adjusted only for race/ethnicity owing to the published evidence of higher adjusted TBI mortality in Hispanic veterans, showed a significant association of Hispanic ethnicity (HR=1.08; 95% CI=1.03–1.13) with higher mortality risk compared with non-Hispanic White ethnicity. The second model, adjusted for veteran location only, showed the U.S. territory location (HR=2.00; 95% CI=1.88–2.13) was significantly associated with higher mortality risk compared with urban mainland location. The third model, adjusted for race/ethnicity and veteran location, also showed that the U.S. territory location (HR=2.10; 95% CI=1.95–2.25) was significantly associated with higher mortality risk compared with urban mainland location, and Hispanic ethnicity ceased to be associated with a higher risk of mortality compared with non-Hispanic White ethnicity. The fourth model adjusted for TBI severity, sociodemographic as well as military and comorbidity covariates, also showed that the U.S. territory location was associated with a higher risk of mortality (HR=1.24; 95% CI=1.15–1.34) relative to urban mainland location. In addition, moderate TBI (HR=1.41; 95% CI=1.35–1.47) and penetrating TBI (1.39; 95% CI=1.25–1.55) but not severe TBI were significantly associated with higher adjusted mortality relative to mild TBI. Age 35–50 (HR=2.04; 95% CI=1.84–2.25), age 51–61 (HR=3.71; 95% CI=3.35–4.10), and age 62 and older (HR=9.67; 95% CI=8.74–10.70) were significantly associated with higher mortality relative to age 18–34. The higher mortality risk for the U.S. territory veterans was also apparent in the Kaplan–Meier survival curve for time to death by veteran geographic location as given in Figure 1.

**Table 2.** **Adjusted Mortality Using Cox Proportional Hazard: Geographic Disparities in the U.S. Veteran Traumatic Brain Injury Mortality, January 1, 2000 to December 31, 2014**

	Model 1 (race/ethnicity), HR (95% CI)	Model 2 (location), HR (95% CI)	Model 3 (location, demographics, TBI severity), HR (95% CI)	Model 4 (location, demographics, TBI severity comorbidities), HR (95% CI)
Location				
Mainland urban		Reference	Reference	Reference
Mainland rural		0.98 (0.95–1.01)	0.92^a^ (0.90–0.95)	0.97^a^ (0.94–1.00)
Mainland highly rural		0.91 (0.80–1.03)	0.85^a^ (0.75–0.96)	0.87^a^ (0.77–0.98)
U.S. territories		2.00^a^ (1.88–2.13)	2.10^a^ (1.95–2.25)	1.24^a^ (1.15–1.34)
Race/ethnicity				
Non-Hispanic White	Reference		Reference	Reference
Non-Hispanic Black	0.62^a^ (0.60–0.65)		0.61^a^ (0.59–0.64)	0.62^a^ (0.59–0.65)
Hispanic	1.08^a^ (1.03–1.13)		0.86^a^ (0.82–0.91)	0.86^a^ (0.81–0.92)
Other race	0.61^a^ (0.56–0.67)		0.58^a^ (0.53–0.63)	0.86^a^ (9.79–0.94)
Age categories				
Age 18–34				Reference
Age 35–50				2.04^a^ (1.84–2.25)
Age 51–61				3.71^a^ (3.35–4.10)
Age 62 and older				9.67^a^ (8.74–10.70)
Female				0.63^a^ (0.59–0.68)
OEF/OIF veteran				0.70^a^ (0.60–0.80)
Combat veteran				0.69^a^ (0.60–0.80)
Any service connected disability				0.96^a^ (0.93–0.99)
Married				0.83^a^ (0.81–0.86)
TBI severity				
Mild TBI				Reference
Moderate TBI				1.41^a^ (1.35–1.47)
Severe TBI				1.07 (0.98–1.17)
Penetrating TBI				1.39^a^ (1.25–1.55)
Unknown TBI severity				1.05 (0.99–1.12)

The sample size was 114,593 veterans with an *ICD-9* code for TBI served by the Veterans Health Administration between January 1, 2000 and December 31, 2010 and followed until death or December 31, 2014. Model 4 included binary indicators of the 31 Elixhauser Comorbidities which are not shown in the table. U.S. territories include Puerto Rico, U.S. Virgin Islands, Guam, America Samoa, and Mariana Islands.

^a^ *p*<0.05.

CI, confidence interval; HR, hazard ratio.

## Discussion

The purpose of this study was to examine the longitudinal associations among race and ethnicity, geographic location, and all-cause mortality in veterans with TBI. Our findings indicated that veterans residing in the U.S. territories had lower median survival compared with veterans residing in the U.S. mainland. To our knowledge, this is the first study to compare TBI-related mortality rates among veterans by geographic location that included the U.S. territories. The sequential models indicated that the U.S. territory veteran location was associated with higher mortality risk among veterans clinically diagnosed with TBI. This relationship may account for the published evidence of Hispanic ethnicity being associated with higher mortality risk, given the high proportion of Hispanic veterans living in the U.S. territories. The findings regarding rural areas contrast with much of previous research suggesting that rural residents have poorer outcomes regarding chronic health conditions compared with their urban peers.^16,17,19,20^ Instead, our findings showed little difference in the mean survival months between rural and urban veterans, but significant differences between those residing in the U.S. territories. Our findings are consistent with the results of one study that suggested that, after controlling for demographic factors (i.e., age, education, and zip-code median income), rural veterans at age 65 had a lower mortality rate from all causes than their urban peers.^24^ Based on these results geography should be considered a vulnerability factor, as opposed to a specific type of geography, and disadvantage may exist in rural, urban, or the U.S. territories based on the specific area in question.

These findings underscore the fact that tacking health disparities is not a one size fits all process, and tailored approaches are necessary to address, and eliminate gaps. We found that veterans residing in the U.S. territories tended to be 62 years or older, Hispanic, have moderate TBI, and have a higher median number of comorbid conditions compared with their mainland peers. When compared with veterans residing in urban mainland areas, veterans seeking care in the U.S. territories had a higher risk of mortality. The reasons for higher mortality rates within this population remain unclear. It is possible that veterans in the U.S. territories have worse overall health, given older age and prevalence of comorbid conditions; however, the excess mortality remains after adjusting for these factors. These findings may be a result of differential utilization, as previous research suggests that Hispanic veterans were less likely to utilize TBI services, including specialty clinics, neurology, and rehabilitation services in the VA health care system.^12,14^ Low service utilization could lead to condition exacerbation or failure to identify preventable medical conditions early and before becoming chronic.

Taken together, the results of this study indicated that for veterans, residing in a U.S. territory was associated with higher TBI mortality risk. Considering that Puerto Rico accounts for 90% of the total population in the U.S. territories,^25^ it is critical that researchers learn more about the variations in provision of care that could contribute to higher TBI-related mortality. These findings suggest that there may be several social and demographic factors related to location of residence that contribute to ethnic differences in chronic condition mortality. One study that compared nonfederal hospitals located in the U.S. mainland with hospitals in the U.S. territories discharging Medicare fee-for-service patients with a principal discharge diagnosis of acute myocardial infarction, heart failure, or pneumonia found that the U.S. territory hospitals reported higher risk-standardized 30-day mortality rates than hospitals in the U.S. mainland.^26^ This study also found disparities in compliance with a set of guideline-recommended therapies for acute myocardial infarction, heart failure, and pneumonia.^26^ Such findings raise concerns regarding potential differences in health care quality between the U.S. mainland and U.S. territories. It is possible that the U.S. veterans residing in the U.S. territories may also not be receiving similar appropriate TBI rehabilitation and other treatment as mainland U.S. veterans diagnosed with TBI. To date, little research has examined this topic, with focus on actionable results to initiate change in populations experiencing disparities.

This study has several limitations. First, we included only veterans who had clinically confirmed diagnoses of TBI during a 10-year period. Second, the current analyses are limited because of the absence of data collection on several contextual factors that could contribute to elevated mortality rates, including cause of death, time between TBI injury date and entry into the VA, TBI mechanism of injury, coinsurance or cotreatment at facilities outside the VA health care system, Glasgow Coma Score (the gold standard for TBI severity), discharge disposition, education level, degree of family support, and degree of disability at the time of death. Relatedly, the data set did not include information on the quality of health care in the U.S. territories; therefore, we were unable to identify factors that may have further exacerbated TBI-related mortality within this population.

### Implications for health equity

In this study, we sought to determine whether geographic location accounted for the association between race/ethnicity and TBI-related mortality risk among veterans clinically diagnosed with TBI after controlling for relevant covariates. Our findings suggest that residence in the U.S. territories is an important factor and may help explain higher mortality for Hispanic veterans given the high proportion of Hispanics living in the U.S. territories. Given the scarcity of published research in this area, the reasons regarding this novel finding remain unknown. Future research is required to better understand the associations among location, mortality, and TBI among veterans residing in the U.S. territories. Specifically, further research needs to compare TBI rehabilitation and other treatment between the U.S. territories and U.S. mainland veterans diagnosed with TBI. Finally, the U.S. Department of Veterans Affairs has a mission of ensuring equitable treatment of all veterans and should investigate targeted policies and interventions to improve the survival of the U.S. territory veterans diagnosed with TBI.

## Abbreviations Used

CIs: confidence intervals
HRs: hazard ratios
OEF: Operation Enduring Freedom
OIF: Operation Iraqi Freedom
RUCA: Rural-Urban Commuting Areas
TBI: traumatic brain injury
VA: Veterans Affairs
VINCI: VA Informatics and Computing Infrastructure
VHA: Veterans Health Administration
